# Examining the Relationship Between Physical Function and Anxiety/Depression in Parkinson's

**DOI:** 10.1002/brb3.70563

**Published:** 2025-05-26

**Authors:** Philip Hodgson, Alastair Jordan, Charikleia Sinani, Divine Charura

**Affiliations:** ^1^ Physiotherapy Department Tees, Esk and Wear Valleys NHS Foundation Trust, West Park Hospital Darlington UK; ^2^ School of Science, Technology and Health York St John University York UK; ^3^ School of Education, Language and Psychology York St John University York UK

**Keywords:** mental health, Parkinson's disease, physical health, physiotherapy, symptom interaction

## Abstract

**Background:**

Parkinson's disease (PD) is a complex neurological disorder characterized by both motor and nonmotor symptoms, including tremor, muscle stiffness, anxiety, and depression.

**Objectives:**

The primary aim of this study was to examine the relationship between physical function and psychological symptoms, specifically anxiety and depression, in people with Parkinson's (PwP). The secondary aim was to explore whether any discrepancies between participant‐reported and clinician‐rated measures of physical function exist.

**Methods:**

This study utilized the Parkinson's Progression Markers Initiative (PPMI) dataset, analyzing data from 1065 individuals with PD. Correlational analyses assessed relationships between clinician‐rated and participant‐reported motor outcomes alongside psychological symptoms. Multiple linear regression (MLR) was employed to identify predictors of anxiety and depression.

**Results:**

In PwP, significant correlations were found between depression/anxiety and participant‐reported motor function (via MDS‐UPDRS Part II: *r* = 0.313 for depression, *r* = 0.284 for anxiety, *p* < 0.05). In contrast, correlations with clinician‐rated motor function (via MDS‐UPDRS Part III) were weaker (*r* = 0.079 for depression, *p* < 0.05; *r* = 0.054 for anxiety, *p* = 0.08). MLR analysis indicated that in PwP, age, cognition, and participant‐reported motor function explained 11.2% of the variance in depression and 10.5% in anxiety.

**Conclusions:**

This study highlights a discrepancy between psychological symptoms and their relationship with clinician‐rated versus participant‐reported motor function in PwP. Our findings suggest that factors such as age, cognitive level, and perceived physical function significantly influence this relationship. Consequently, it is crucial to consider psychological factors and participant‐reported motor function when conducting clinical assessments and treatment planning for individuals with PD.

## Graphical abstract

This study reveals significant correlations between participant‐reported motor function and psychological symptoms of anxiety and depression in Parkinson's disease. Weaker associations were found between anxiety/depression and clinician‐rated measures of physical function. Factors such as age and cognition appear to influence these relationships, emphasizing the importance of integrating patient perspectives in treatment planning. This approach can inform assessment and referral practices, potentially enhancing patient‐centered care and improving outcomes.

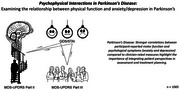

## 1 Introduction

Parkinson's disease (PD) is a progressive neurodegenerative disorder primarily affecting older adults (Bloem et al. 2021). It is characterized by bradykinesia, resting tremor, rigidity, and various nonmotor symptoms, including sleep disturbances and mood disorders (Rana et al. 2015). Currently, over 10 million people worldwide are living with PD (Prasad and Hung 2021), with its prevalence having doubled in the last 25 years, indicating a significant public health concern (Ou et al. 2021).

Motor symptoms such as tremor, bradykinesia, and rigidity hinder daily activities and independence, while nonmotor symptoms—including cognitive impairment, mood disorders, sleep disturbances, and gastrointestinal issues—also detract from quality of life (Candel‐Parra et al. 2021; Gökçal et al. 2017; Kurihara et al. 2020). Studies show that increased anxiety correlates with worsened motor symptoms as measured by the Unified Parkinson's Disease Rating Scale (UPDRS) (Zahodne et al. 2013; Stefanova et al. 2013; Dissanayaka et al. 2010; Brown et al. 2011), suggesting a complex interplay between psychological and physical health. The relationship between these factors remains unclear beyond UPDRS measures (Lutz et al. 2016; Leentjens et al. 2012). Meta‐regression analyses indicate a strong correlation between depressive symptoms and impaired physical function, particularly regarding mobility and balance (Hodgson et al. 2024). As PD progresses, complications from long‐term medication use and worsening symptoms exacerbate these issues (Gómez‐Esteban et al. 2007), underscoring the importance of comprehensive assessment and management strategies that address both motor and nonmotor symptoms to improve the overall quality of life for people with Parkinson's (PwP).

Patient‐reported outcome measures (PROMs) and clinician‐rated outcome measures (CROMs) serve as complementary methods for evaluating PD (Churruca et al. 2021), including monitoring condition progression and treatment efficacy. PROMs capture subjective experiences related to quality of life and symptom severity (Churruca et al. 2021), while CROMs provide objective assessments of physical and cognitive function (Zdravkovic et al. 2022). Comparing these assessments helps to provide a more comprehensive understanding of a condition's impact on patients (Zdravkovic et al. 2022). Discrepancies between clinical findings and the perception of PwP may reveal hidden issues regarding aspects that may not emerge during stand‐alone evaluations (Zdravkovic et al. 2022). Integrating both perspectives can promote patient‐centered care and improve communication between PwP and healthcare providers, leading to tailored treatment plans that address the aspects of PD most important to the recipients of clinical care (Engle et al. 2021; Bloem et al. 2020; Molzahn and Northcott 1989).

Understanding discrepancies between patient perceptions and clinical assessments is crucial for evaluating psychological impacts on physical function. For instance, in conditions such as rheumatoid arthritis, patients often report greater disability than clinicians recognize, which can lead to underestimation of their experiences (Sacristán et al. 2020). Similar mismatches have been noted in poststroke patients, particularly those with depression (Essers et al. 2021). In PD, where depressive symptoms are common, such discrepancies may exist where patients’ reports on physical function may not align with clinician assessments. Identifying the reasons behind any discrepancies could inform better clinical practices.

Current guidelines, such as the European Physiotherapy Guideline for Parkinson's Disease (Keus et al. 2019), primarily emphasize clinician‐rated measures over PROMs. This oversight limits the understanding of patients’ perceptions of their physical function and the psychological symptoms affecting their well‐being. By neglecting PROMs, clinicians might miss crucial insights that could enhance assessment practices and inform clinical decisions.

This study aims to address this gap by examining the relationship between physical function and psychological symptoms, specifically anxiety and depression, in PwP. The secondary aim was to explore whether any discrepancies between participant‐reported and clinician‐rated measures of physical function exist. We hypothesized a significant association between physical function measures and the severity of anxiety and depression. Additionally, we anticipated identifying discrepancies between participant‐reported outcomes and clinician‐rated assessments of physical function.

## 2 Methods

### 2.1 Ethical Approval

Ethical approval was obtained via York St John University, School of Science, Technology and Health, prior to undertaking data analysis (Reference: ETH2324‐0171).

### 2.2 Data Source and Selection

The Parkinson's Progression Markers Initiative (PPMI) dataset was used in this research. PPMI is an observational, international, multicenter study designed to identify biomarkers of PD progression. The study was launched in 2010 and monitors participants over time. PPMI collects comprehensive clinical, imaging, and biological data from participants at clinical sites worldwide. The dataset used in our analysis included 1065 individuals with PD. Due to differences in the number of follow‐up assessments for participants, only baseline data for each individual were included in our analysis.

In this analysis, we included individuals at various stages of PD, from early to advanced, as classified by the Hoehn and Yahr (H&Y) scale (stages 1–5). No specific exclusion criteria were applied based on participant characteristics or co‐morbidities. Only participants with complete demographic data and complete data for key variables of interest, such as measures of physical function (both participant‐reported and clinician‐rated) and measures of psychological symptoms, were included in our analysis.

Data concerning physical function, psychological symptoms, cognition, and quality of life were extracted from the PPMI dataset. Measures of physical function included MDS‐UPDRS Part II, MDS‐UPDRS Part III, and Hoehn & Yahr Score (H&Y). Measures of psychological symptoms included the 15‐item Geriatric Depression Scale (GDS) and the State‐Trait Anxiety Inventory (STAI). Other outcomes included in our analysis were the Montreal Cognitive Assessment (MoCA), age, and time since diagnosis. These measures were selected to gauge depression, anxiety, and aspects of motor and cognitive ability alongside more general measures of condition progression.

### 2.3 Statistical Analysis

Data cleaning was performed to identify and handle outliers and erroneous entries, which were subsequently edited in cases of clear misentry or removed where resolution was not possible. Normality tests were conducted on continuous variables, and appropriate nonparametric tests were used where necessary. Descriptive statistics were calculated for all variables, including means, standard deviations, and frequencies.

To assess bivariate relationships between physical function measures (both participant‐reported and clinician‐rated) and psychological symptom scores (GDS for depression and STAI for anxiety), Pearson's correlation coefficients were computed. Comparison of the correlation coefficients involving participant‐reported and clinician‐rated measures provided an insight into the association between different pairs of outcomes.

The correlation coefficient between MDS‐UPDRS Parts II and III assisted in evaluating whether changes in one part are associated with changes in the other, providing an insight into the agreement between participant‐reported and clinician‐rated measures of physical function. A Bland–Altman plot was created to visualize the agreement between the two types of assessments. As the maximum score for Parts II and III is different, to quantify the magnitude of the difference between MDS‐UPDRS Part II and Part III, these measures were standardized using *z*‐scores, and Cohen's *d* was calculated to give an indication of effect size. To further compare the correlations between participant‐reported/clinician‐rated measures of physical function and measures of anxiety/depression, Fisher's *z*‐transformation was used. This process converts Pearson correlation coefficients (*r*) into a normally distributed variable (*z*), facilitating statistical inference.

Multiple linear regression (MLR) models were employed to examine the independent associations between physical function measures and psychological symptoms while controlling for variables such as age, cognition, condition severity, and duration. The backward method was used to identify the most appropriate model by sequentially removing the least significant variables until only significant predictors remained. This allowed us to determine the independent associations between the physical function measures (MDS‐UPDRS Parts II and III) and psychological symptoms (GDS and STAI) while controlling for the other variables. This approach provides insights into the complex relationships between motor and nonmotor symptoms in PD.

All statistical analyses were performed using IBM SPSS Statistics (version 29), with a significance level set at *p* < 0.05. This diverse array of statistical methods facilitated a thorough examination of the relationships between physical function and psychological symptoms.

## 3 Results

Of the 2347 participants included in the PPMI December 2023 data cut, which included healthy control and prodromal groups, 1065 PwP were selected for further analysis. The main outcomes of interest were H&Y Scale, MoCA, GDS, STAI, and MDS‐UPDRS Parts II and III. A summary of participant demographics and outcomes can be seen in Tables 1 and 2a, respectively. Table 2b provides further details on the proportion of individuals reaching the threshold for predefined severity levels for each of the key outcomes.

**TABLE 1:** Demographics.

	Mean		*SD*
Age (years)	62.62		9.86
Time since symptom onset (years)	2.91		3.12
Time since diagnosis (years)	1.33		1.72
BMI	26.93		5.06
Years of education	15.86		3.47
	Category	Count (*n*)	Percentage (%)
Sex	Female	411.00	38.59%
	Male	654.00	61.41%
Race	White	987.00	92.68%
	Black	17.00	1.60%
	Asian	14.00	1.31%
	Other	39.00	3.66%
	Unknown	8.00	0.75%
Family Hx PD	Yes	373.00	35.02%
	No	692.00	64.98%
	Unknown	0.00	0.00%
Handedness	Right	936.00	87.89%
	Left	100.00	9.39%
	Mixed	28.00	2.63%
	Unknown	1.00	0.09%

**TABLE 2a:** Outcomes.

	Mean	*SD*
H&Y (NHY_ON)	1.68	0.51
MOCA	26.66	2.71
GDS	2.53	2.79
STAI	65.51	19.09
TUG^a^	10.81	3.26
Motor Function *Q* ^a^	6.06	2.91
Neuro QoL Lower Extremity^a^	38.39	2.59
Neuro QoL Upper Extremity^a^	38.23	3.02
MDS‐UPDRS Part I Score^a^	6.49	4.86
MDS‐UPDRS Part II Score	6.47	4.73
MDS‐UPDRS Part III Score OFF (includes OFF and untreated scores)^a^	22.48	10.22
MDS‐UPDRS Part III Score ON (includes ON and untreated scores)	21.40	10.08
MDS‐UPDRS Part IV Score^a^	1.76	2.74
MDS‐UPDRS Total Score OFF (includes OFF and untreated scores)^a^	35.35	15.52
MDS‐UPDRS Total Score ON (includes ON and untreated scores)^a^	34.39	15.15

^a^ Incomplete data (TUG, *n* = 15; Motor Function *Q*, *n* = 389; Neuro QoL Lower Extremity, *n* = 389; Neuro QoL Upper Extremity, *n* = 389; MDS‐UPDRS Part I Score, *n* = 1059; MDS‐UPDRS Part III Score OFF [includes OFF and untreated scores], *n* = 1001; MDS‐UPDRS Part IV Score, *n* = 238; MDS‐UPDRS Total Score OFF [includes OFF and untreated scores], *n* = 995; MDS‐UPDRS Total Score ON [includes ON and untreated scores], *n* = 1059).

**TABLE 2b:** Outcomes.

Score	*N*	%
H&Y (NHY_ON) 0 1 2 3	1 366 675 23	0.09 34.37 63.38 2.16
MOCA Normal cognitive performance (≥26) Mild impairment (18–25) Moderate impairment (10–17) Severe impairment (0–9)	768 288 9 0	72.11 27.04 0.85 0
GDS Normal (0–4) Mild depression (5–8) Moderate depression (9–11) Severe depression (12–15)	878 132 42 13	82.44 12.39 3.94 1.22
STAI No or low anxiety (40–75) Moderate anxiety (76–89) High anxiety (90–160)	777 154 134	72.96 14.46 12.58
MDS‐UPDRS Part II Score Mild/moderate (0–12) Moderate (13–29) Severe (30–52)	936 127 2	87.89 11.92 0.19
MDS‐UPDRS Part III Score ON (includes ON and untreated scores) Mild (0–32) Moderate (33–58) Severe (59–132)	901 162 2	84.60 15.21 0.19

### 3.1 Correlation Coefficients

H&Y stage over time (*r* = 0.171, *p* < 0.05) showed an overall worsening of PD severity following diagnosis. Symptoms of anxiety and depression were correlated significantly (*r* = 0.692, *p* < 0.05), showing that these symptoms are closely related. Depression was significantly correlated with participant‐reported motor function via MDS‐UPDRS Part II (*r* = 0.313, *p* < 0.05), whereas the correlation between depression and clinician‐rated function via MDS‐UPDRS Part III was also significant yet weaker (*r* = 0.079, *p* < 0.05). Anxiety significantly correlated with participant motor function via MDS‐UPDRS Part II (*r* = 0.283, *p* < 0.05); however, the correlation between anxiety and clinician‐rated function via MDS‐UPDRS Part III (*r* = 0.054, *p* = 0.08) was not significant. Overall, there was a significant correlation between the participant‐reported measure of physical function (MDS‐UPDRS Part II) and clinician‐rated motor function (MDS‐UPDRS Part III) (*r* = 0.403, *p* < 0.05).

A correlation matrix table for all outcomes of interest can be seen in Table 3.

**TABLE 3:** Correlation matrix.

	H&Y	MoCA	GDS	STAI	UPDRS Part II	UPDRS Part III	Time since diagnosis
MoCA	−0.125^*^						
GDS	0.078^*^	−0.111^*^					
STAI	0.041	−0.101^*^	0.692^*^				
UPDRS Part II	0.297^*^	−0.111^*^	0.313^*^	0.283^*^			
UPDRS Part III	0.533^*^	−0.099^*^	0.079^*^	0.054	0.403^*^		
Time since diagnosis	0.171^*^	−0.116^*^	0.079^*^	0.107^*^	0.211^*^	−0.035	
Time since symptom onset	0.147^*^	−0.056	0.088^*^	0.076^*^	0.169^*^	0.049	0.585

Abbreviations: GDS = Geriatric Depression Scale; H&Y = Hoehn and Yahr Scale; MoCA = Montreal Cognitive Assessment; STAI = State‐Trait Anxiety Inventory; UPDRS = Unified Parkinson's Disease Rating Scale.

*p < 0.05.

Fisher's *z*‐transformation was used to compare the correlation coefficients between participant‐ and clinician‐rated measures of physical function and measures of anxiety/depression. *Z*‐scores of the relevant correlations were compared and are summarized in Table 4.

**TABLE 4:** Comparison of correlation coefficients.

Variable pair	Correlation coefficient (*r*)	Correlation coefficient *Z*‐score (*z_r_*)	Standard error (*SE*)	*Z*‐statistic (*z*)	Probability (*p*)
GDS × MDS UPDRS Part II	0.313	0.323	0.043	5.63	<0.001
GDS × MDS UPDRS Part III	0.079	0.079			
STAI × MDS UPDRS Part II	0.283	0.291	0.043	5.47	<0.001
STAI × MDS UPDRS Part III	0.054	0.054			

Abbreviations: GDS = Geriatric Depression Scale; STAI = State‐Trait Anxiety Inventory; UPDRS = Unified Parkinson's Disease Rating Scale.

The correlation between GDS and MDS‐UPDRS Part II was significantly stronger than the correlation between GDS and MDS‐UPDRS Part III, with a *z*‐statistic of 5.63 (*p* < 0.001). Similarly, the correlation between STAI and MDS‐UPDRS Part II was significantly stronger than that between STAI and MDS‐UPDRS Part III, with a *z*‐statistic of 5.47 (*p* < 0.001).

To offer further insight into the difference between MDS‐UPDRS Parts II and III, Cohen's *d* was calculated. These results indicated a large effect size (*d* = 0.83), suggesting a substantial difference between MDS‐UPDRS Part II and III scores. This result underscores the difference between participant‐reported and clinician‐rated motor function as measured by the MDS‐UPDRS, suggesting that further investigation may be beneficial to understand the clinical implications of these differences.

**FIGURE 1:**
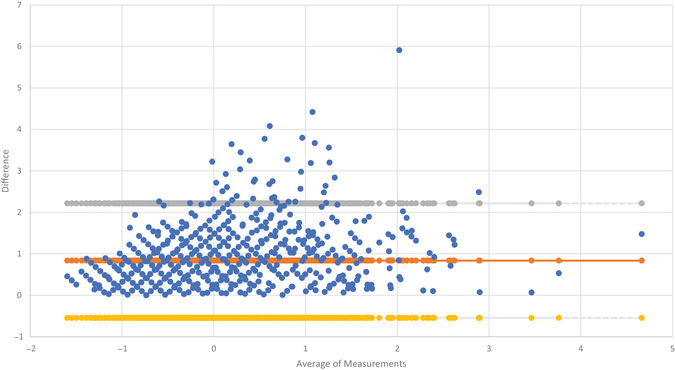
Bland–Altman plot for agreement between MDS‐UPDRS Parts II and III.

A Bland–Altman plot (Figure 1) displays the agreement between MDS‐UPDRS Parts II and III. Scatter trends indicate that a proportional bias may exist, with the difference between measurements increasing with the magnitude of measurements. Most points lie within the 95% agreement limits. On further examination, outliers with a large difference between outcomes tended to consist of higher scores for MDS‐UPDRS Part II in comparison to Part III score. This indicates that these individuals perceive their level of physical function to be worse in comparison to the assessment completed by a clinician.

### 3.2 MLR

To determine the independent associations between physical function measures and psychological symptoms while controlling for age, cognition, condition severity, and duration, we conducted MLR analyses using the backward method. The dependent variables (DVs) were measures of psychological symptoms, specifically the GDS and STAI. The independent variables (IVs) included age, time since symptom onset/diagnosis, H&Y stage, MoCA score, and MDS‐UPDRS Parts II and III.

The results of the MLR analyses indicate that both the GDS and STAI models were statistically significant (*p* < 0.001). The models explain 11.2% and 10.5% of the variance in their respective outcomes (*R* ^2^ = 0.112 and *R* ^2^ = 0.105). For GDS, significant predictors include age, which has a negative effect (*B* = −0.066, *p* = 0.030); cognition (via MoCA), which also has a negative effect (standardized *B* = −0.094, *p* = 0.002); and MDS‐UPDRS Part II, which shows a significant positive effect (*B* = 0.325, *p* < 0.001). Other variables, including time since symptom onset, time since diagnosis, H&Y stage, and MDS‐UPDRS Part III, did not significantly impact GDS. In the STAI model, age again demonstrated a significant negative effect (*B* = −0.121, *p* < 0.01), and MoCA has a significant negative effect (*B* = −0.096, *p* = 0.001), whereas MDS‐UPDRS Part II showed a significant positive effect (*B* = 0.294, *p* < 0.001). Similar to the GDS analysis, time since symptom onset, time since diagnosis, H&Y stage, and MDS‐UPDRS Part III did not significantly influence STAI scores. These findings highlight the importance of age, cognitive function, and perceived physical disability in predicting psychological symptoms in PwP.

## 4 Discussion

Current guidelines, such as the European Physiotherapy Guideline for Parkinson's Disease (Keus et al. 2019), which aims to support decision‐making for physiotherapy practice in managing PD, primarily emphasize clinician‐rated measures over PROMs. Similarly, insufficient attention is given to the integration of PROMs that reflect the subjective experiences of PwP (Zolfaghari et al. 2022). This gap in research knowledge limits our understanding of how psychological symptoms, such as anxiety and depression, may influence or be influenced by patients’ perceptions of their physical function.

The study utilized existing data from the PPMI with the aim of examining the relationship between physical function and psychological symptoms, specifically anxiety and depression, in PwP. Key findings highlight significant correlations between measures of physical function and psychological symptoms, specifically depression and anxiety, independent of whether these were participant‐reported outcomes or clinician‐rated. Overall, this study demonstrates the importance of considering both clinician‐rated and patient‐reported outcomes in the assessment of PD. While clinician‐rated measures provide valuable objective data, patient‐reported outcomes capture the subjective experience of living with PD, offering insights into the impact of the condition on daily life, mental health, and well‐being. The potential discrepancies observed between these two perspectives may serve as indicators, prompting clinicians to investigate underlying psychological symptoms such as anxiety and depression.

Our analysis revealed a correlation between measures of physical function and psychological symptoms. Higher levels of physical impairment, as measured by the MDS‐UPDRS Parts II and III, were associated with higher scores on the GDS and the STAI. These correlations were more prominent for the participant‐reported measures of physical function (via MDS‐UPDRS Part II) in comparison to clinician‐rated measures of physical function (via MDS‐UPDRS Part III). This suggests that an individual's perceptions of their own motor function may play a more critical role in their psychological well‐being than previously acknowledged.

To quantify the influence of factors such as age, cognition, condition severity, and duration, MLR analyses were conducted. The results showed that the relationship between physical function and psychological symptoms remained significant even after controlling for these variables, highlighting the independent role of perceived physical function in psychological well‐being in PD. Notably, participant‐reported motor function scores, such as those from the MDS‐UPDRS Part II, contributed to a significant worsening of psychological measures such as GDS and STAI scores, while clinician‐rated assessments from MDS‐UPDRS Part III did not. This suggests that an individual's perception of their motor function is crucial for their psychological health, indicating that healthcare providers and clinical guidelines should prioritize PROMs alongside CROMs.

Consistent with existing literature (Still et al. 2021), our study demonstrates significant correlations between physical impairments and psychological distress, which are stronger in participant‐reported outcomes in comparison to clinician‐rated measures. This reinforces the associated decline in motor function alongside worsening psychological symptoms.

Previous research has established that motor symptoms, such as rigidity and bradykinesia, are often accompanied by increased rates of depression and anxiety (Lacy et al. 2023; Papapetropoulos et al. 2006; Starkstein et al. 1990). The current study adds to this body of evidence by utilizing a large and diverse dataset from the PPMI, which includes participants at various stages of PD. Within this dataset, 187 individuals (17.56%) scored ≥5 on the GDS, indicating the likely presence of depression. This is comparable to results from previous research (Baillon et al. 2013; Goodarzi et al. 2016). However, when specifically considering severe depression (scores ≥12), only 13 individuals (1.22%) met this threshold; therefore, this sample may not be fully representative of the PD population experiencing more severe depression. Our comprehensive approach allows for a more nuanced understanding of how physical function relates to psychological health across different stages of PD. This new evidence provides insight into how perceived physical function relates to psychological symptoms of anxiety and depression. In the context of symptoms of anxiety and depression, our MLR regression models suggest that the impact of perceived physical function may be more prominent than the impact of motor symptoms as assessed by clinicians. Our insight emphasizes the importance of incorporating patient perspectives into the evaluation of their health status, which can help to generate more tailored treatment strategies.

Our research corroborates existing findings and also provides new insights that can inform clinical practice. By emphasizing the usefulness of PROMs in addition to CROMs, the study advocates for a more integrated approach to managing PD, where both physical rehabilitation and psychological support are prioritized. This holistic perspective is essential for improving patient outcomes and advancing the understanding of the complex interplay between physical and psychological health in PD.

The study reveals that while both assessment methods are associated with psychological symptoms, they may capture different aspects of a patient's experience. PROMs often reflect the individual's perception of their functional abilities and limitations in daily activities, which can be influenced by their psychological state and previous functional ability. In contrast, CROMs provide a current, objective evaluation of motor symptoms from the perspective of clinicians. This divergence can significantly impact patient care and treatment strategies. For instance, if clinicians rely solely on their assessments, they may overlook critical insights from patients regarding their perceived functional limitations and psychological distress. Therefore, integrating both perspectives into clinical practice may lead to more personalized treatment plans that focus on addressing not only the physical but also the psychological needs of PD patients, ultimately improving their quality of life and treatment outcomes.

We suggest that declines in motor abilities may exacerbate psychological distress, while psychological factors may also influence perceptions of physical function. This challenges traditional models that often treat physical and psychological symptoms as separate entities, advocating instead for a more integrated framework that considers how these domains interact. Furthermore, the study's emphasis on the differences between participant‐reported and clinician‐rated measures underscores the importance of subjective experiences in understanding the overall impact of PD. This insight encourages researchers and clinicians to adopt a holistic approach in both research and treatment, recognizing that addressing psychological symptoms may be as crucial as managing motor symptoms to improve patient outcomes. Ultimately, these findings advance the theoretical discourse on PD by promoting a comprehensive understanding of the complex interplay between physical and psychological health, paving the way for more effective interventions and support strategies.

### 4.1 Limitations

This study has limitations that must be acknowledged when interpreting the findings. One limitation is the potential for selection bias, as the PPMI dataset consists of participants who are willing and able to engage in a long‐term study. This may exclude individuals with more severe PD or comorbid conditions who are unable to participate, potentially skewing the results toward those with less severe manifestations of PD, including psychological symptoms. Furthermore, the representativeness of the sample may be limited, as the PPMI cohort may not fully capture the diversity of the broader PD population, including variations in ethnicity, socioeconomic status, and access to health care, limiting generalizability. Reporting bias may influence participant‐reported measures, as individuals may either overestimate or underestimate their symptoms based on personal perceptions or recall inaccuracies. Clinician measurement bias is also a potential concern, as measures may be subject to the evaluator's subjective judgment. Given the nature of the above limitations within an existing dataset, we were unable to remove all limitations.

While our study provides an overview of the relationships between physical function and psychological symptoms of anxiety and depression at a single point in time, it does not capture potential individual‐level changes or developments in these relationships over the course of the disease. Despite this, our included participants provide representation of individuals at varying times since their individual diagnoses.

While the MDS‐UPDRS and psychological assessments such as the GDS and STAI are well‐established, they may not capture the full spectrum of physical function and psychological symptoms experienced by PwP. The reliance on self‐reporting for psychological symptoms can introduce variability based on individual perspectives, while clinician assessments may not fully reflect the patient's functional capabilities in real‐world settings. These limitations necessitate cautious interpretation of the results and suggest that future research should aim to incorporate a more diverse sample and utilize a broader range of assessment tools to enhance the validity and generalizability of findings.

Finally, the MDS‐UPDRS is designed to assess PD‐specific symptoms. This does therefore not permit meaningful comparison with healthy control and/or prodromal groups, as these populations do not exhibit the same PD‐specific symptoms. This limitation emphasizes the necessity for future research to incorporate non‐PD‐specific outcomes, which would enable more meaningful comparisons across diverse groups and populations. By broadening the scope of assessment, a more comparable understanding of the experiences and challenges faced by individuals at various stages of PD will be possible.

### 4.2 Future Research

Our research findings suggest several key areas for future investigation to enhance the understanding of PD and address current study limitations. First, expanding the diversity of the study population is crucial, as the PPMI dataset may not adequately reflect the broader PD population's heterogeneity in ethnicity, socioeconomic status, and healthcare access. A more diverse cohort would improve the generalizability of findings regarding the interaction between physical function and psychological symptoms across different demographic groups.

Additionally, future research should prioritize longitudinal analyses to explore how these relationships evolve over time as the condition progresses, providing insights that could inform targeted interventions. Developing and validating more comprehensive measurement tools for both physical function and psychological symptoms is also essential. This should include PROMs that capture subjective experiences and CROMs that reflect clinical perspectives. Incorporating qualitative methods, such as interviews and focus groups, could further illuminate patients’ lived experiences and coping strategies.

Future research should aim to develop holistic assessment tools encompassing both objective and subjective aspects of physical function and psychological symptoms, enhancing the understanding of the complex interplay between motor and nonmotor symptoms in PD. This knowledge is crucial for improving the quality of life for patients, as it contributes to a growing body of research focused on cognitive and psychological aspects of PD. While the study has limitations, such as potential selection bias and the representativeness of the sample, addressing these in future research could lead to improved assessment methods and targeted interventions.

Overall, this study advances our understanding of PD's multifaceted impact on patients’ lives, advocating the need for a comprehensive, patient‐centered approach to both research and care, thereby informing future studies aimed at better outcomes for individuals living with PD.

## Author Contributions

**Philip Hodgson**: conceptualization, methodology, funding acquisition, validation, formal analysis, project administration, writing–original draft, writing–review and editing, visualization. **Alastair Jordan**: methodology, resources, supervision, writing–review and editing. **Charikleia Sinani**: methodology, resources, writing–review and editing, supervision. **Divine Charura**: methodology, resources, writing—review and editing, supervision.

## Disclosure

The authors declare that there are no additional disclosures to report.

## Ethics Statement

Ethical approval was obtained via York St John University, School of Science, Technology and Health, prior to undertaking data analysis (Reference: ETH2324‐0171). Informed patient consent was not necessary for this work. We confirm that we have read the Journal's position on issues involved in ethical publication and affirm that this work is consistent with those guidelines.

## Conflicts of Interest

The authors declare no conflicts of interest.

### Peer Review

The peer review history for this article is available at https://publons.com/publon/10.1002/brb3.70563

## Data Availability

Data sharing is not applicable to this article as no new data were created or analyzed in this study.
